# Supplementary material for: Artificially decreasing cortical tension generates aneuploidy in mouse oocytes
Source: Nat Commun. 2020 Apr 3;11:1649. doi: 10.1038/s41467-020-15470-y (PMC7125192; doi:10.1038/s41467-020-15470-y)
Supplement: Supplementary file 1 — Supplementary Information [file 41467_2020_15470_MOESM1_ESM.pdf]

Supplementary Information

Artificially decreasing cortical tension generates aneuploidy in mouse oocytes

Bennabi et al.

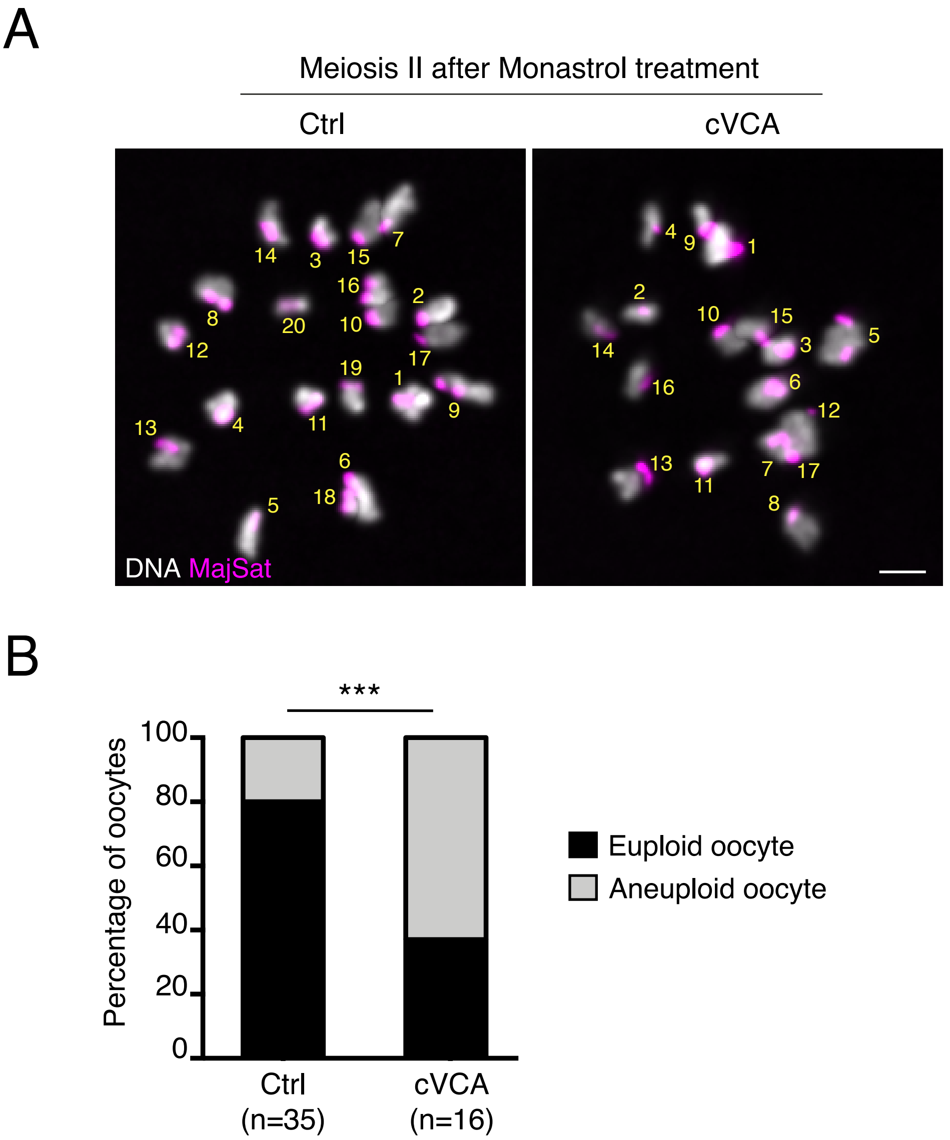

Supplementary Figure 1: Extra-soft oocytes are aneuploid.

(A) Z-projections from control and cVCA oocytes in meiosis II incubated 2h in 200 µM Monastrol, expressing Histone(H2B)-GFP (grey) and MajSat-mClover (magenta). Scale bar: 5 µm. The chromosomes are arbitrarily numbered in yellow. This experiment was repeated independently 3 times with similar results. **(B)** Bar graph representing the percentage of euploid oocytes (black) and aneuploid oocytes (grey) in meiosis II for controls and cVCA oocytes. (n) is the number of oocytes analyzed. Data are from 3 independent experiments. Statistical significance of differences is assessed with a Chi-square test two-sided: ***P-value<0.0001.

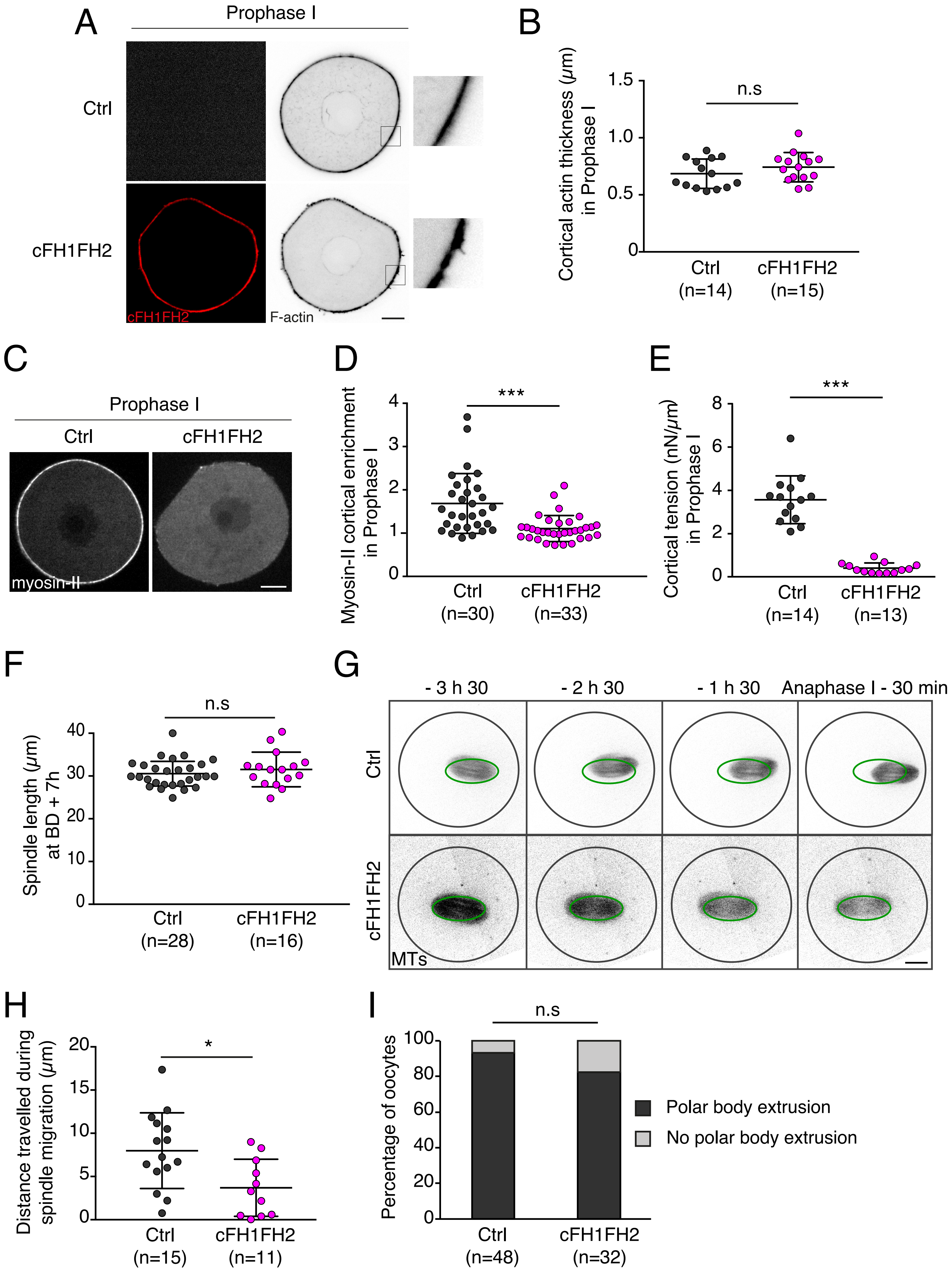

A
Prophase I
Ctrl
cFH1FH2
cFH1FH2
F-actin
B
Cortical actin thickness (μm) in Prophase I
n.s
1.5
1.0
0.5
0
Ctrl (n=14)
cFH1FH2 (n=15)
C
Prophase I
Ctrl
cFH1FH2
myosin-II
D
Myosin-II cortical enrichment in Prophase I
***
Ctrl (n=30)
cFH1FH2 (n=33)
E
Cortical tension (nN/μm) in Prophase I
***
Ctrl (n=14)
cFH1FH2 (n=13)
F
Spindle length (μm) at BD + 7h
n.s
Ctrl (n=28)
cFH1FH2 (n=16)
G
- 3 h 30
- 2 h 30
- 1 h 30
Anaphase I - 30 min
Ctrl
cFH1FH2
MTs
H
Distance travelled during spindle migration (μm)
*
Ctrl (n=15)
cFH1FH2 (n=11)
I
Percentage of oocytes
n.s
Polar body extrusion
No polar body extrusion
Ctrl (n=48)
cFH1FH2 (n=32)

Supplementary Figure 2: cFH1FH2 oocytes also display a decrease in cortical tension and an absence of spindle migration.

(A) Representative images of control and cFHFH2 (red) Prophase I oocytes expressing GFP-UtrCH (black). Right insets show magnifications of the cortex. Scale bar: 10 µm. 2 independent experiments. **(B)** Cortical actin thickness in Prophase I control and cFH1FH2 oocytes. Data are from 2 independent experiments. (n) is the number of oocytes analyzed. Statistical significance was tested with a t-test two-sided: n.s P-value=0.24. **(C)** Representative images of control and cFH1FH2 oocytes expressing SF9-GFP (grey) in Prophase I. Scale bar: 10 µm. 2 independent experiments. **(D)** Dot plot representing myosin-II cortical enrichment (see Methods) for control and cFH1FH2 oocytes. (n) is the number of oocytes analyzed. Data are from 2 independent experiments. Statistical significance of differences is assessed with a Mann-Whitney test two-sided: ***P-value<0.0001. **(E)** Dot plot showing cortical tension values in control and cFH1FH2 oocytes (see Methods). (n) is the number of oocytes analyzed. Data are from 3 independent experiments. Statistical significance of differences is assessed with a Mann-Whitney test two-sided: *** P-value <0.0001. **(F)** Spindle length for controls and cFH1FH2 oocytes 7 hours after nuclear envelope breakdown. (n) is the number of oocytes analyzed. Data are from 4 independent experiments. Statistical significance is determined with a Mann-Whitney test two-sided: n.s P-value=0.4066. **(G)** Representative time-lapse movies showing spindle (SiR-Tubulin, black) position in a control and a cFH1FH2 oocyte. The green ovals mark initial spindle positions. Acquisitions were taken every 30 minutes starting 3h30 before anaphase I. Scale bar: 10 µm. 5 independent experiments. **(H)** Dot plot showing the distance travelled by the spindle starting 4h before anaphase I for control and cFH1FH2 oocytes. Data are from 5 independent experiments. (n) is the number of oocytes analyzed. Statistical significance of the differences was assessed with a t-test two-sided: * P-value=0.012. **(I)** Rate of polar body extrusion in control and cFH1FH2 oocytes. Data are from 4 independent experiments. (n) is the number of oocytes analyzed. Statistical significance tested with a Fisher's test two-sided: n.s P-value=0.1871. For (B, D, E, F, H) black bars and whiskers represent mean and standard deviation.

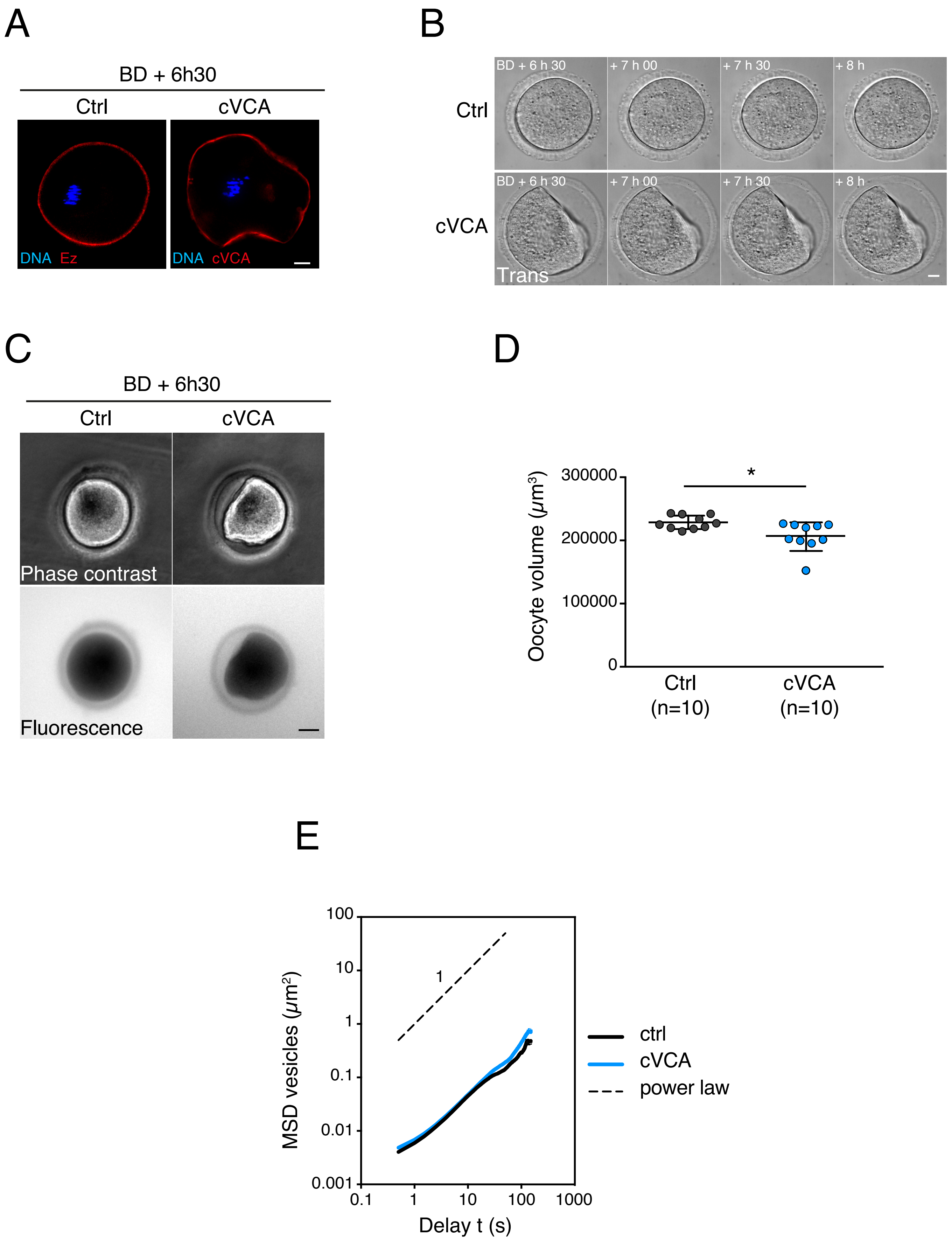
A
BD + 6h30
Ctrl
cVCA
DNA Ez
DNA cVCA
B
Ctrl
cVCA
BD + 6 h 30
+ 7 h 00
+ 7 h 30
+ 8 h
Trans
C
BD + 6h30
Ctrl
cVCA
Phase contrast
Fluorescence
D
Oocyte volume (µm³)
300000
200000
100000
0
*
Ctrl
(n=10)
cVCA
(n=10)
E
MSD vesicles (µm²)
100
10
1
0.1
0.01
0.001
0.1
1
10
100
1000
Delay t (s)
1
ctrl
cVCA
power law

Supplementary Figure 3: Cell volume but not cytoplasmic activity is reduced in extra-soft oocytes.

(A) Confocal spinning disk images of control and cVCA oocytes at BD + 6h30. Chromosomes are labeled with Histone(H2B)-GFP (blue). The cortex is labeled with Ezrin-mCherry in control and Ezrin-mCherry-cVCA in cVCA oocytes (both red). Scale bar: 10 µm. This experiment was repeated independently 3 times with similar results. (**B**) Representative time-lapse movies of a control and a cVCA oocyte showing that oocytes are stable in shape between BD + 6h30 and BD + 8h. Acquisitions were taken every 30 minutes. Scale bar: 10 µm. This experiment was repeated independently 3 times with similar results. **(C)** For volume measurements, control and cVCA oocytes are in chambers filled with fluorescent dextran (bottom panels). Scale bar: 20 µm. This experiment was repeated independently 3 times with similar results. **(D)** Oocyte volume is reduced in cVCA oocytes compared to controls. Each dot represents the volume (μm^3) of one oocyte from 3 independent experiments. (n) is the number of oocytes analyzed. Black bars and whiskers represent mean and standard deviation. A Wilcoxon rank sum test two-sided was performed to compare the mean (black line) of the two conditions: *P-value=0.0232. **(E)** The motion of Nile red-labeled vesicles is similar in cVCA oocytes compared to controls, suggesting comparable cytoplasmic activity. The dashed line represents a power law of 1. MSD data are fitted to a simple linear regression model (R^2>0.97). Data are from 3 independent experiments.

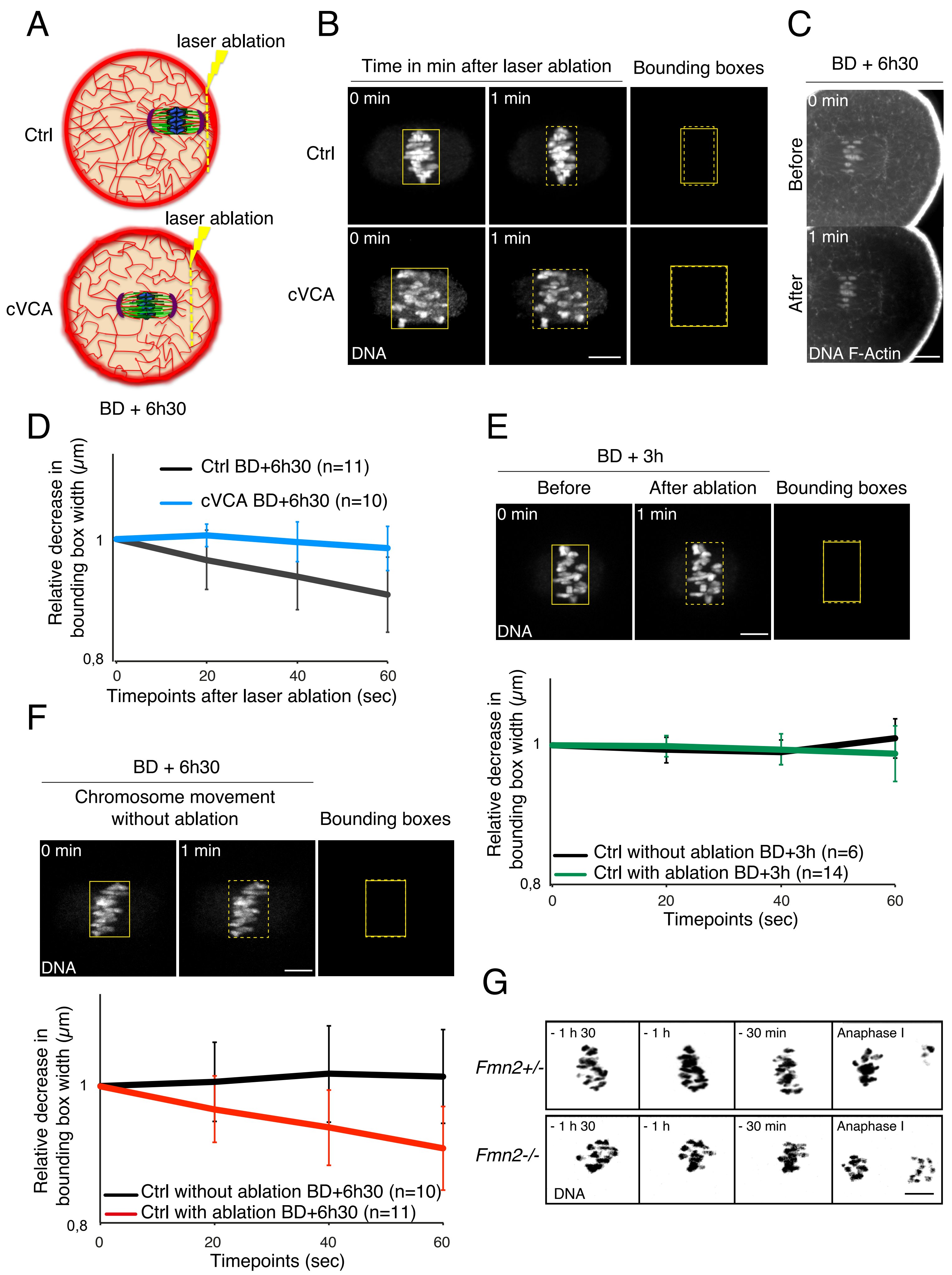
A
laser ablation
Ctrl
laser ablation
cVCA
BD + 6h30
B
Time in min after laser ablation
Bounding boxes
0 min
1 min
Ctrl
0 min
1 min
cVCA
DNA
C
BD + 6h30
0 min
Before
1 min
After
DNA F-Actin
D
Relative decrease in bounding box width (µm)
Ctrl BD+6h30 (n=11)
cVCA BD+6h30 (n=10)
1
0,8
0
20
40
60
Timepoints after laser ablation (sec)
E
BD + 3h
Before
After ablation
Bounding boxes
0 min
1 min
DNA
Relative decrease in bounding box width (µm)
1
0,8
Ctrl without ablation BD+3h (n=6)
Ctrl with ablation BD+3h (n=14)
0
20
40
60
Timepoints (sec)
F
BD + 6h30
Chromosome movement without ablation
Bounding boxes
0 min
1 min
DNA
Relative decrease in bounding box width (µm)
1
0,8
Ctrl without ablation BD+6h30 (n=10)
Ctrl with ablation BD+6h30 (n=11)
0
20
40
60
Timepoints (sec)
G
- 1 h 30
- 1 h
- 30 min
Anaphase I
Fmn2+/-
- 1 h 30
- 1 h
- 30 min
Anaphase I
Fmn2-/-
DNA

Supplementary Figure 4: The forces applied to the chromosomes are altered in extra-soft oocytes but it is not the main cause of chromosome misalignment.

(A) Scheme of the laser ablation in control and cVCA oocytes. Actin is in red, microtubules in green, chromosomes in blue and myosin-II in purple. **(B)** Representative images at BD + 6h30 of oocytes expressing Histone(H2B)-GFP and EB3-GFP (grey) in control and cVCA oocytes. Bounding boxes of the metaphase I plate are represented in solid lines before and dashed lines 1 min after ablation. Scale bar: 10 µm. 5 independent experiments. **(C)** Laser ablation (before and 1 minute after) of F-actin between the spindle and the closest cortex at BD + 6h30 in a control oocyte expressing Histone(H2B)-GFP (grey) and GFP-UtrCH (also in grey). Scale bar: 10 µm. 5 independent experiments. **(D)** Graph representing the relative decrease of the metaphase I plate bounding boxes every 20 seconds (s) during 1 minute in control and cVCA oocytes. (n) is the number of oocytes analyzed. Data are compiled from 5 independent experiments. **(E)** Representative images at BD + 3h of a control oocyte expressing Histone(H2B)-GFP and EB3-GFP (grey). Bounding boxes of the chromosomes are represented in solid lines before and dashed lines 1 min after ablation. Scale bar: 10 µm. The graph below represents the relative decrease of the chromosomes bounding boxes at BD + 3h with or without laser ablation. (n) is the numbers of oocytes analyzed. **(F)** Representative images at at BD + 6h30 of a control oocyte expressing Histone(H2B)-GFP and EB3-GFP (grey). Bounding boxes of the metaphase I plate are represented in solid lines and dashed lines 1 min after. Scale bar: 10 µm. The graph below represents the relative decrease of the metaphase I plate bounding boxes at BD + 6h30 without laser ablation or following ablation (red). (n) is the numbers of oocytes analyzed. **(G)** Representative time-lapse movies of control and *Fmn2-/-* oocytes expressing Histone(H2B)-GFP (black). Acquisitions were taken every 30 minutes starting 1h30 before anaphase I. Scale bar: 10 µm. 3 independent experiments. For (D, E, F), the mean and standard deviation are shown for each timepoint.

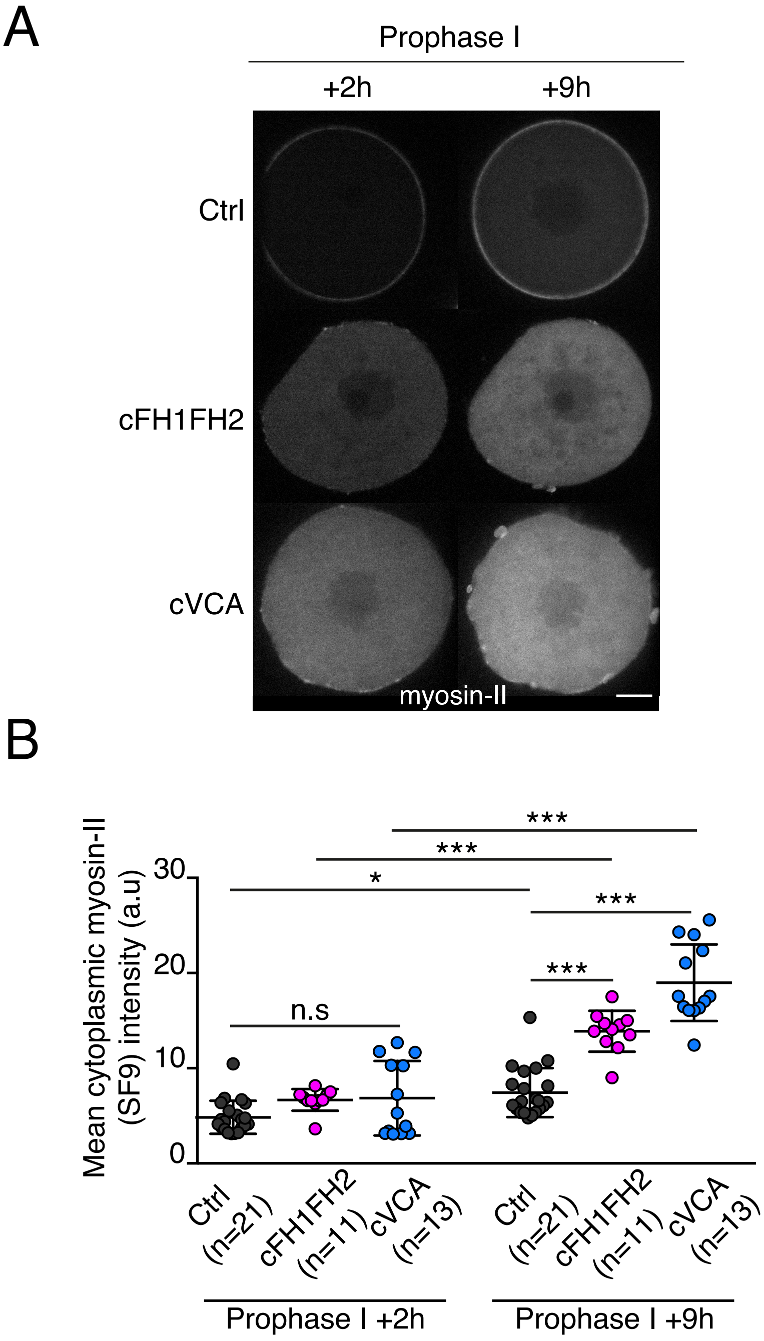

Supplementary Figure 5: Myosin-II accumulates in the cytoplasm in extra-soft oocytes over time.

(A) Confocal spinning disk images of control, cVCA and cFH1FH2 oocytes expressing SF9-GFP (myosin-II intrabody, grey). Prophase I oocytes are observed 2h and 9h after cRNA injection. Scale bar: 10 µm. This experiment was repeated independently 3 times with similar results. **(B)** Dot plot representing the SF9-GFP cytoplasmic intensity in prophase I arrested oocytes 2h and 9h after expression of SF9-GFP alone (control) or together with cVCA or cFH1FH2. Six measurements were taken in the cytoplasm for each oocyte. (n) is the number of oocytes analyzed. Data are from 3 independent experiments. Black bars and whiskers represent mean and standard deviation. Statistical significance of differences is assessed with a One-Way ANOVA test. For the Prophase I +2h groups, all conditions (Ctrl vs. cFH1FH2, Ctrl vs. cVCA, cVCA vs. cFH1FH2) are all n.s P-value>0.9999. Regarding the evolution of SF9-GFP cytoplasmic intensity, Ctrl Prophase I +2h vs. +9h are different (*P-value=0.0335), and cFH1FH2 Prophase I +2h vs. +9h and cVCA Prophase I +2h vs. +9h are different (***P-values<0.0001).
