# Supplementary material for: Artificially decreasing cortical tension generates aneuploidy in mouse oocytes
Source: Nat Commun. 2020 Apr 3;11:1649. doi: 10.1038/s41467-020-15470-y (PMC7125192; doi:10.1038/s41467-020-15470-y)
Supplement: Supplementary file 3 — Description of Additional Supplementary Files [file 41467_2020_15470_MOESM3_ESM.pdf]

Description of Additional Supplementary Files

File Name: Supplementary Movie 1
Description: Z-stack (z-steps of 500 nm) in meiosis II 2h after incubation in 200 �M Monastrol from confocal images of a control (left) and an extra-soft cVCA oocyte (right) expressing Histone(H2B)-GFP (grey) to label chromosomes and MajSat-mClover (magenta) to label the Major satellite repeats. Related to FigS1A.

File Name: Supplementary Movie 2
Description: High-frequency time-lapse movies of a control (top) and extra-soft cVCA oocyte (bottom) in transmitted light (left) expressing Histone(H2B)-GFP together with either Ez-mCherry or cVCA-mCherry (right, both in grey). The movies start at BD + 6h30 with acquisitions taken every 500 mseconds for 5 minutes. The first 75 frames are shown.

File Name: Supplementary Movie 3
Description: High-frequency time-lapse movies of a control (top) and extra-soft cVCA oocyte (bottom) incubated in Nile Red at BD + 6h30 with acquisitions taken every 500 mseconds for 5 minutes. The first 75 frames are shown. Related to FigS3E.

File Name: Supplementary Movie 4
Description: Time-lapse movies of a control (top) and extra-soft cVCA oocyte (bottom) expressing Histone(H2B)-GFP to label chromosomes and EB3-GFP to label the spindle (both in grey) at BD + 6h30, first timepoint before laser ablation, then acquisitions taken every 20 seconds during 1 minute. Related to FigS4B.

File Name: Supplementary Movie 5
Description: Time-lapse movie of a control oocyte expressing Histone(H2B)-GFP to label chromosomes and GFP-UtrCH to label F-actin (both in grey) at BD + 6h30, first timepoint before laser ablation, then acquisitions taken every 20 seconds during 1 minute. Related to FigS4C.

File Name: Supplementary Movie 6
Description: Time-lapse movie of a control oocyte expressing Histone(H2B)-GFP to label chromosomes and EB3-GFP to label the spindle (both in grey) at BD + 3h, first timepoint before laser ablation, then acquisitions taken every 20 seconds during 1 minute. Related to FigS4E.

File Name: Supplementary Movie 7
Description: Time-lapse movie of a control oocyte expressing Histone(H2B)-GFP to label chromosomes and EB3-GFP to label the spindle (both in grey) at BD + 6h30. Acquisitions were taken every 20 seconds during 1 minute. Related to FigS4F.

File Name: Supplementary Movie 8
Description: Time-lapse movies of spindle morphogenesis in a control (left) and extra-soft cVCA oocyte (right, cVCA in red) expressing Histone(H2B)-GFP (blue) to label chromosomes and incubated with SiR-Tubulin to label microtubules (green). Z-projections, 10 sections of 4 µm every 1h. The movies start 1h after NEBD and last until anaphase I with acquisitions every hour. Related to Fig2A.

File Name: Supplementary Movie 9
Description: Time-lapse movies of monoasters in a control (left) and extra-soft cVCA oocyte (right) expressing EB3-GFP (black) to label MT plus-tips and incubated with Monastrol to avoid spindle bipolarization. One Z-plane, every 250 ms. The movies start 2h after NEBD. Related to Fig2B and C.

File Name: Supplementary Movie 10
Description: Time-lapse movies of a control (left) and extra-soft cVCA oocyte (right) expressing Histone(H2B)-GFP (grey) to label chromosomes. The movies start at BD + 6h30 with acquisitions every 20 seconds for 20 minutes. Related to Fig3A.

File Name: Supplementary Movie 11
Description: Time-lapse movies of a control (left) and extra-soft cVCA oocyte (right) expressing Histone(H2B)-GFP (blue) to label chromosomes. The movies start at NEBD with acquisitions every 30 minutes until BD + 6h30. The magenta ellipses circle the chromosomes. Related to Fig3C.

File Name: Supplementary Movie 12
Description: Z-stack (z-steps of 500 nm) at BD + 6h30 from time-lapse movies of a control (left) and extra-soft cVCA oocyte (right) expressing Histone(H2B)-GFP (grey) to label chromosomes and MajSat-mClover (magenta) to label the Major satellite repeats. Related to Fig3D.
