# Supplementary material for: Artificially decreasing cortical tension generates aneuploidy in mouse oocytes
Source: Nat Commun. 2020 Apr 3;11:1649. doi: 10.1038/s41467-020-15470-y (PMC7125192; doi:10.1038/s41467-020-15470-y)
Supplement: Supplementary file 16 — Reporting Summary [file 41467_2020_15470_MOESM16_ESM.pdf]

naturereresearch

Corresponding author(s): MH VERLHAC and ME TERRET

Last updated by author(s): Feb 24, 2020

Reporting Summary

Nature Research wishes to improve the reproducibility of the work that we publish. This form provides structure for consistency and transparency in reporting. For further information on Nature Research policies, see Authors & Referees and the Editorial Policy Checklist .

Statistics

For all statistical analyses, confirm that the following items are present in the figure legend, table legend, main text, or Methods section.

n/a	Confirmed	
☐	☒	The exact sample size (*n*) for each experimental group/condition, given as a discrete number and unit of measurement
☐	☒	A statement on whether measurements were taken from distinct samples or whether the same sample was measured repeatedly
☐	☒	The statistical test(s) used AND whether they are one- or two-sided<br>*Only common tests should be described solely by name; describe more complex techniques in the Methods section.*
☒	☐	A description of all covariates tested
☐	☒	A description of any assumptions or corrections, such as tests of normality and adjustment for multiple comparisons
☐	☒	A full description of the statistical parameters including central tendency (e.g. means) or other basic estimates (e.g. regression coefficient) AND variation (e.g. standard deviation) or associated estimates of uncertainty (e.g. confidence intervals)
☐	☒	For null hypothesis testing, the test statistic (e.g. *F*, *t*, *r*) with confidence intervals, effect sizes, degrees of freedom and *P* value noted<br>*Give P values as exact values whenever suitable.*
☒	☐	For Bayesian analysis, information on the choice of priors and Markov chain Monte Carlo settings
☒	☐	For hierarchical and complex designs, identification of the appropriate level for tests and full reporting of outcomes
☒	☐	Estimates of effect sizes (e.g. Cohen's *d*, Pearson's *r*), indicating how they were calculated

Our web collection on statistics for biologists contains articles on many of the points above.

Software and code

Policy information about availability of computer code

Data collection	Metamorph Software (Universal Imaging) version 7.7.9.0 was used to collect data.
Data analysis	Matlab version 2016a, Metamorph version 7.7.9.0, ImageJ and Fiji (NIH) version 2.0.0-rc-69/1.52, Microsoft Excel version 16.16.19, GraphPad Prism 8 and R Version 3.3.2 were used to analyze the data.

For manuscripts utilizing custom algorithms or software that are central to the research but not yet described in published literature, software must be made available to editors/reviewers. We strongly encourage code deposition in a community repository (e.g. GitHub). See the Nature Research guidelines for submitting code & software for further information.

Data

Policy information about availability of data

All manuscripts must include a data availability statement. This statement should provide the following information, where applicable:

- Accession codes, unique identifiers, or web links for publicly available datasets
- A list of figures that have associated raw data
- A description of any restrictions on data availability

All relevant data supporting the finding of this study are available from the corresponding author upon request.

Field-specific reporting

Please select the one below that is the best fit for your research. If you are not sure, read the appropriate sections before making your selection.

☒ Life sciences ☐ Behavioural & social sciences ☐ Ecological, evolutionary & environmental sciences

For a reference copy of the document with all sections, see nature.com/documents/nr-reporting-summary-flat.pdf

October 2018

Life sciences study design

All studies must disclose on these points even when the disclosure is negative.

Sample size	Experiments were repeated at least 3 times, n numbers were 10 or more, with very few variability (see s.d) and the adequate statistic tests were performed. No predetermination of sample size were performed. The sample sizes (oocytes number for each experiment) were chosen based on the number of mature follicles of mice.
Data exclusions	No data were excluded from the analysis.
Replication	Experiments were repeated at least 3 times, n numbers were 10 or more, with very few variability (see s.d). Ali attempts at replication were successful.
Randomization	When oocytes were collected from different mice from similar strains, they were allocated into experimental groups randomly before microinjection.
Blinding	Investigators were blinded for data analysis.

Reporting for specific materials, systems and methods

We require information from authors about some types of materials, experimental systems and methods used in many studies. Here, indicate whether each material, system or method listed is relevant to your study. If you are not sure if a list item applies to your research, read the appropriate section before selecting a response.

Materials & experimental systems

n/a	Involved in the study	
☐	☒	Antibodies
☒	☐	Eukaryotic cell lines
☒	☐	Palaeontology
☐	☒	Animals and other organisms
☒	☐	Human research participants
☒	☐	Clinical data

Methods

n/a	Involved in the study	
☒	☐	ChIP-seq
☒	☐	Flow cytometry
☒	☐	MRI-based neuroimaging

Antibodies

Antibodies used	rabbit anti-Phospho-MLC2 (Ser19) (Cell Signaling # 3671; 1:200), Alexa-594-labeled anti-rabbit (Invitrogen # A-21207; 1:400).
Validation	The rabbit anti-Phospho-MLC2 (Ser19) antibody was already described and used in mouse oocytes (Dumont et al., 2007; Schuh et al., 2008). In addition, the staining disappears after the inhibition of the activation of the protein (the antibody recognizes the active form of the protein) as shown in the manuscript.

Animals and other organisms

Policy information about studies involving animals; ARRIVE guidelines recommended for reporting animal research

Laboratory animals	11-week-old OFI, fmn2-/- or fmn2+/- (Leader et al., 2002) female mice were used, housed in the animal facility of the CIRB.
Wild animals	No wild animais were used for this study.
Field-collected samples	No field-collected samples were used for this study.
Ethics oversight	All experimental procedures used for the project have been approved by the ministry of agriculture to be conducted in our animal facility (authorization N°75-1170). The use of all the genetically modified organisms described in this project has been granted by the DGRI (Direction Générale de la Recherche et de l'Innovation: Agrément OGM; DUO-1783).

Note that full information on the approval of the study protocol must also be provided in the manuscript.
